# Assessment of the Efficacy of Circulating Tumor Cells by Liquid Biopsy in the Diagnosis and Prediction of Tumor Behavior of Gliomas: A Systematic Review

**DOI:** 10.7759/cureus.54101

**Published:** 2024-02-13

**Authors:** Teena Eugene, Jano Roy SG, Nivethitha S, Meethu Rappai

**Affiliations:** 1 Pathology, SRM Medical College Hospital and Research Centre, SRM Institute of Science and Technology (SRMIST), Chennai, IND

**Keywords:** circulating tumor, glioblastoma multiforme, biomarkers, glioblastoma, liquid biopsy, central nervous system

## Abstract

In the realm of glioma management, the ability to accurately diagnose and predict tumor behavior remains a formidable task. Emerging as a beacon of hope, liquid biopsy (LB), with its potential to detect circulating tumor (CT) cells, offers a novel and promising avenue for addressing these challenges. This systematic review delves into the effectiveness of LB in transforming the landscape of glioma analysis as well as prognosis, shedding light on its clinical significance and implications.

We conducted a comprehensive literature search from 2015 to 2023, using multiple sources. We assessed titles and abstracts first, followed by full-text review if they met our criteria. We included those studies that fulfill the inclusion criteria of the study. For bias assessment, we used a two-part tool for specific domains and a quality assessment tool for diagnostic accuracy studies.

In this review, we incorporated eight studies. A total of 498 patients were identified across eight studies. The average sensitivity was 72.28% in seven of these studies, while the average specificity was 91.52% in the same seven studies.

Our review revealed a sensitivity of 72.28% and an impressive specificity of 91.52%. This underscores the potential of LB as a valuable prognostic tool for detecting CT cells. However, the early detection of tumor cells and the prediction of tumor behavior in gliomas continue to be topics of debate, necessitating further research for more precise and reliable outcomes.

## Introduction and background

Gliomas represent the most prevalent primary tumors originating in the central nervous system (CNS). The malignancy grade of gliomas differs across various histotypes, encompassing grades ranging from I to IV [1]. They are characterized by elevated heterogeneity as well as assertiveness, leading to high rates of relapse and mortality [2]. Notably, even with the standard therapeutic regimen involving complete surgical resection ensuingly adjuvant radiotherapy and chemotherapy, the average survival rate is not greater than 15 months from the time of analysis [3,4].

Gliomas constitute 24.7% of all main brain tumors and a significant 74.6% of malignant brain tumors [5]. Among gliomas, glioblastoma multiforme (GBM), the maximum malignant subtype, is categorized by its extremely destructive and infiltrating nature, making it challenging to manage effectively. Current treatment strategies involve surgical resection, subsequently radiotherapy, and temozolomide (TMZ) chemotherapy. However, inadequate drug diffusion into the CNS and the fast expansion of chemotherapy resistance remain persistent challenges, ultimately leading to a poor prognosis [6,7]. Traditionally, tissue biopsies have served as the gold standard for histological as well as molecular tumor investigation, aiding in diagnosis, tumor classification, mutation detection, and treatment planning [8]. However, tissue biopsies are invasive and can pose risks such as bleeding and potential damage to critical brain regions, particularly in pediatric cases [9].

Furthermore, invasive procedures face limitations in attaining adequate as well as high-quality tumor samples. The sequential collection of tissue biopsies for monitoring tumor response and relapse presents a significant task in tumor profiling [10]. This approach often yields insufficient material for conducting all essential molecular tests required for a dependable diagnosis. Consequently, detecting mutations, genetic as well as epigenetic abnormalities, or polymorphisms may not always be feasible in such cases [11].

In light of the limitations posed by conventional biopsies, contemporary oncology research has increasingly turned its attention to the examination of biological fluids as opposed to whole tissue samples in the context of tumor-derived components. This approach is commonly known as a "liquid biopsy" (LB). The term "liquid biopsy" is basically introduced for the analysis of CTCs21 but is now also associated with circulating tumor DNA (ctDNA) and other biomarkers such as microRNAs (miRNAs). Since 2004, the clinical use of LB has increased significantly, when Guardant Health released the first commercially available LB test. During the period of 2016, LB (restricted to ctDNA analysis) was officially adopted as a diagnostic tool in the main reference center laboratories [11].

While blood is the primary source for LBs due to its accessibility to most tumors, it's worth noting that other bodily fluids such as pleural effusions, mucosa, urine, and cerebrospinal fluid (CSF) are also subjected to analysis [12]. Hence, LB offers a heightened level of sensitivity in the diagnostic process, coupled with the advantage of non-invasiveness and the ability for repeated sampling during treatment, offering a much more convenient approach [13]. LB, a minimally invasive procedure, has emerged, advancing diagnostics and providing insights into risk, prognosis, as well as recurrence [14]. Therefore, in the present study, we assess the efficacy of circulating tumor (CT) cells through LB for diagnosing and predicting the behavior of gliomas.

## Review

Methodology

**Figure 1:**
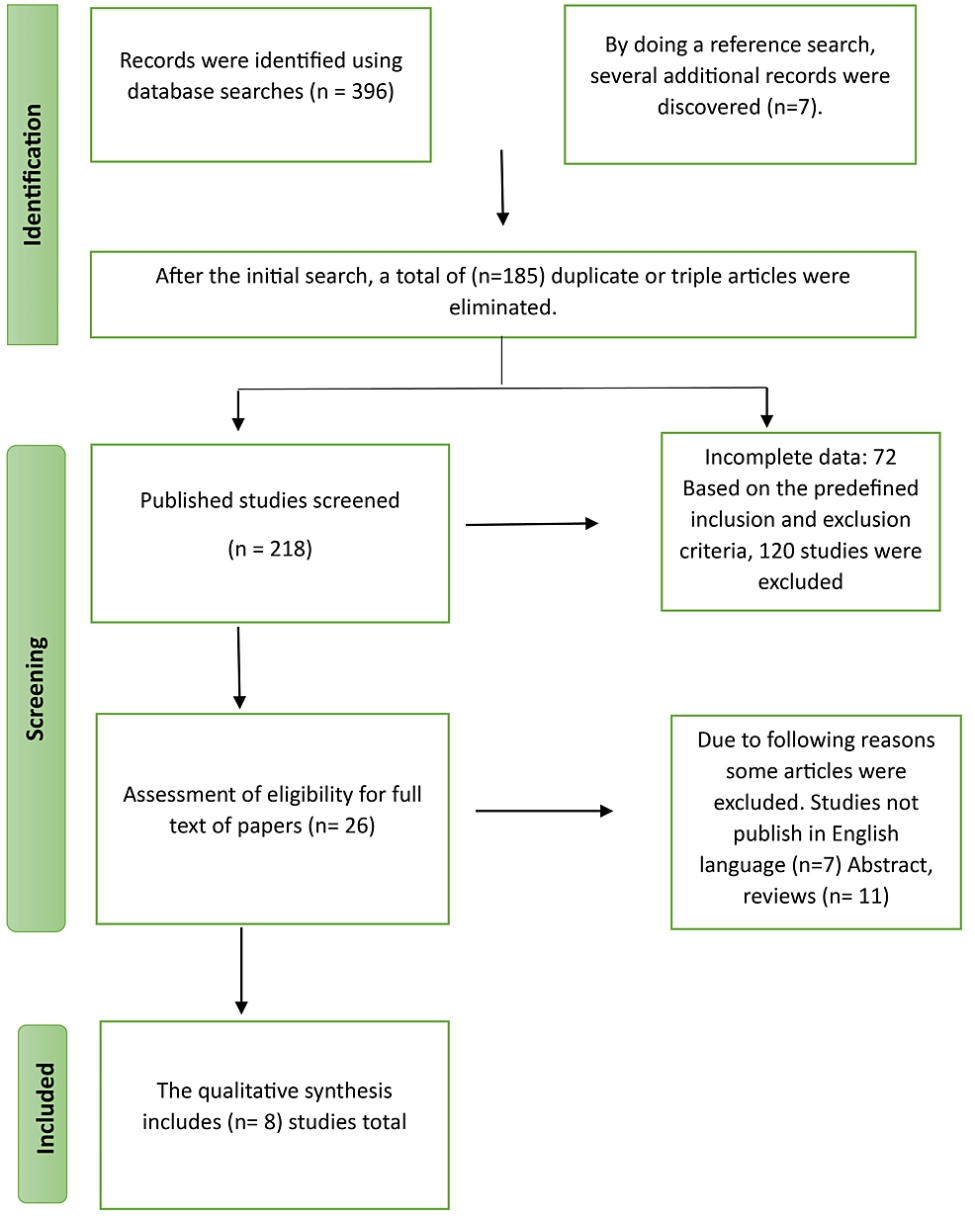
The study assortment criteria for systematic review

A comprehensive literature search was conducted from 2015 to 2023, comprising Medline, Web of Science, PubMed, and additional sources such as Google Scholar and ClinicalTrials.gov. The Preferred Reporting Items for Systematic Reviews and Meta-Analyses (PRISMA) guidelines were used for conducting the review. The following terms were combined for the literature search: "liquid biopsy," "glioma," "prediction of tumor," "tumor behavior," and "glioblastoma." The research methodology commenced with an initial screening process based on the evaluation of titles and abstracts. The complete text of the selected article was assessed for in-depth examination only when the title and abstract met the predetermined inclusion criteria. A breakdown of the search plan is depicted in Figure 1.

Inclusion Criteria

Studies related to gliomas, studies that have biomarker information, and studies stating data in terms of sensitivity, specificity, and literature available in the English language were included.

Exclusion Criteria

Only abstracts, letters, reviews, and studies that are available in a non-English language were excluded.

Statistical analysis

Following the initial search, the selected articles from the database were organized in an Excel spreadsheet, and any duplicate articles were subsequently eliminated. Subsequently, the abstracts and full-text articles were independently reviewed by two authors. The study was conducted by both authors reading all the selected articles, and the final decision was reached collectively (Table 1).

**Table 1:** Features of involved studies GBM: glioblastoma multiforme; HC: healthy control; CSF: cerebrospinal fluid; HGG: high-grade glioma; EGFRvIII: epidermal growth factor receptor variant III; NGS: next-generation sequencing; PCR: polymerase chain reaction; qRT-PCR: quantitative real-time reverse-transcription PCR; EV: extracellular vesicle; LB: liquid biopsy; cfDNA: circulating free DNA

Reference	Total patients	Collected sample	Biomarker	Detection method	Sensitivity	Specificity	Outcomes in the diagnosis of gliomas
[15]	89 GBM	Blood and CSF samples	MGMT	PCR	65%	100%	Successful use of CSF for detecting glioma DNA suggests promising clinical applications, but large-scale trials are needed for validation in diagnosis, treatment planning, recurrence monitoring, and prognosis assessment.
[16]	71	CSF	wtEGFR and EGFRvIII RNA in CSF-derived EVs	PCR	61%	98%	CSF-derived EVs reflect GBM genetic status, aiding in precise EGFRvIII tumor detection and guiding targeted therapies.
[17]	96 HGG, 50 HC	Serum	EGFRvIII RNA in serum-derived EVs	PCR	81.58%	79.31%	The authors have developed a serum-based approach to identify EGFRvIII in high-grade brain tumors. This method has the potential to serve as a non-invasive and efficient diagnostic tool for detecting EGFRvIII-positive HGG.
[18]	20 GBM, 20 HC	Serum	miR-221, miR-222	qRT-PCR	90%	90%	The identification of circulating miR-221 and miR-222 holds promise as a molecular marker for diagnosing and predicting outcomes in GBM patients. It is advisable to conduct additional research with a substantial sample size to further validate these findings.
[19]	95 GBM, 60 HC	Blood	miR-100	qRT-PCR	77.89%	83.33%	The presence of serum miR-100 exhibits promise as a valuable biomarker for both the diagnosis and prognostic assessment of GBM.
[20]	25 GBM, 20 HC	Blood	miR-17-5p, miR-125b, miR-221	qRT-PCR	50.5%, 52.9%, 76.5%	100%, 100%, 100%	Examining miRs in liquid biopsies offers a minimally invasive, stable, and cost-effective way to detect solid tumors, predict treatment response, and monitor patient outcomes.
[21]	52 GBM, 5 anaplastic astrocytomas	Serum	miR-21, miR-222, miR-124-3p	qRT-PCR	-	-	miR-21, miR-222, and miR-124-3p found in serum exosomes can serve as valuable molecular biomarkers to supplement clinical assessments for early tumor progression in cases with HGG during post-surgical therapy.
[22]	25 GBM, 25 HC	Plasma	cfDNA	NGS	80%	90%	LB revolutionizes GBM diagnosis and treatment by providing non-invasive access to mutation data and fusion gene targets, enhancing precision across all disease stages.

Result

The initial search yielded 396 articles, identified through database searches and registrations. Additionally, seven records were found by conducting a reference search. We excluded 185 duplicates and screened 218 records, eliminating 192 due to missing parameters, incompleteness, and inclusion and exclusion criteria. Following the application of inclusion as well as exclusion criteria, we thoroughly evaluated 26 complete articles. Some of these articles contained only abstracts and were not available in English, resulting in their removal. After thorough analysis, we selected eight studies, encompassing data from 2015 to 2023.

Study characteristics

A total of 498 cases were identified across eight studies in this review [15-22]. The average sensitivity was 72.28% in seven of these studies [15-19,22], while the average specificity was 91.52% in the same seven studies [15-19,22] (Table 1).

The majority of studies predominantly concentrated on miRNAs, a class of non-coding RNA molecules renowned for their impact on gene expression. The assessed miRNAs encompassed miR-21, miR-222, miR-124-3p, miR-17-5p, miR-125b, miR-221, as well as miR-100. In all of these investigations, the revealing technique employed was polymerase chain reaction (PCR) analysis accomplished on samples from blood, plasma, or CSF. Three of the studies explored various miRNA types capable of diagnosing glioma cases with varying sensitivity as well as specificity, while three other studies recognized prognostic significance to specific miRNAs. Instead, a study has revealed that the detection of MGMT promoter methylation in CSF samples exhibits greater sensitivity in comparison to serum samples for individuals with glioma [15].

miR-21, miR-222, and miR-124-3p exhibit as a biomarker for the early prognosis of cancer; henceforth, it is proven as a more effective treatment. These indicators provide an efficient method for screening individuals for possible cancers that are yet undetected. If the results are favourable, the tumor's presence is promptly investigated, which permits early intervention and treatment to address the underlying tumor and decrease its possibility of metastasis [23]. Importantly, modulation of miR-21 expression seems to play a broad role in sensitivity to chemotherapy in cancer cells. Notably, increased miR-21 expression has been linked to resistance to platinum-based chemotherapy, as well as to gemcitabine. Furthermore, increased serum miR-21 levels were found to predict a worse response to chemotherapy with trastuzumab [21]. 

The nanoflare was constructed by modifying the antisense single-stranded DNA of miRNA on AuNPs, and the DNAs were prehybridized with Cy5-modified short complementary sequences. The Cy5 groups on nanoflares were quenched in proximity to the AuNPs. The nanoflares can penetrate into the exosomes and replace the Cy5-labeled short sequences with target miRNA hybridization, thus leading to the restoration of the fluorescence intensity detected by a thermophoretic sensor. For the investigation of exosome uptake of AuNPs, Shi et al. used different sizes of DNA-functionalized AuNPs for the uptake efficiency of exosomes and sensing sensitivity of exo-miRNAs [20].

The investigation assessed ctDNA utilizing plasma as the specimen. The integrated analysis of plasma concentration of circulating free DNA (cfDNA), gene mutations, as well as gene-gene fusions has been proposed as a diagnostic approach to differentiate GBM cases that might benefit from directed treatment. This method demonstrated a high level of sensitivity and specificity, with values of 80% and 90%, respectively [22]. Notably, this study is unique in that it employed next-generation sequencing (NGS) as the detection method.

Two studies examined the potential of extracellular vesicles (EVs) as biomarkers for high-grade glioma (HGG) by focusing on the assessment of epidermal growth factor receptor variant III (EGFRvIII) status [16,17]. A serum-based technique for EGFRvIII assessment in HGG offers a promising non-invasive analytical technique [17]. CSF-derived EVs in GBM cases carry an RNA signature that reflects both wtEGFR expression and EGFRvIII status. In the initial study, approximately 2-3 mL of serum volume was employed for RNA isolation per specimen, while the second study utilized 1 mL [16].

Risk of bias

**Figure 2:**
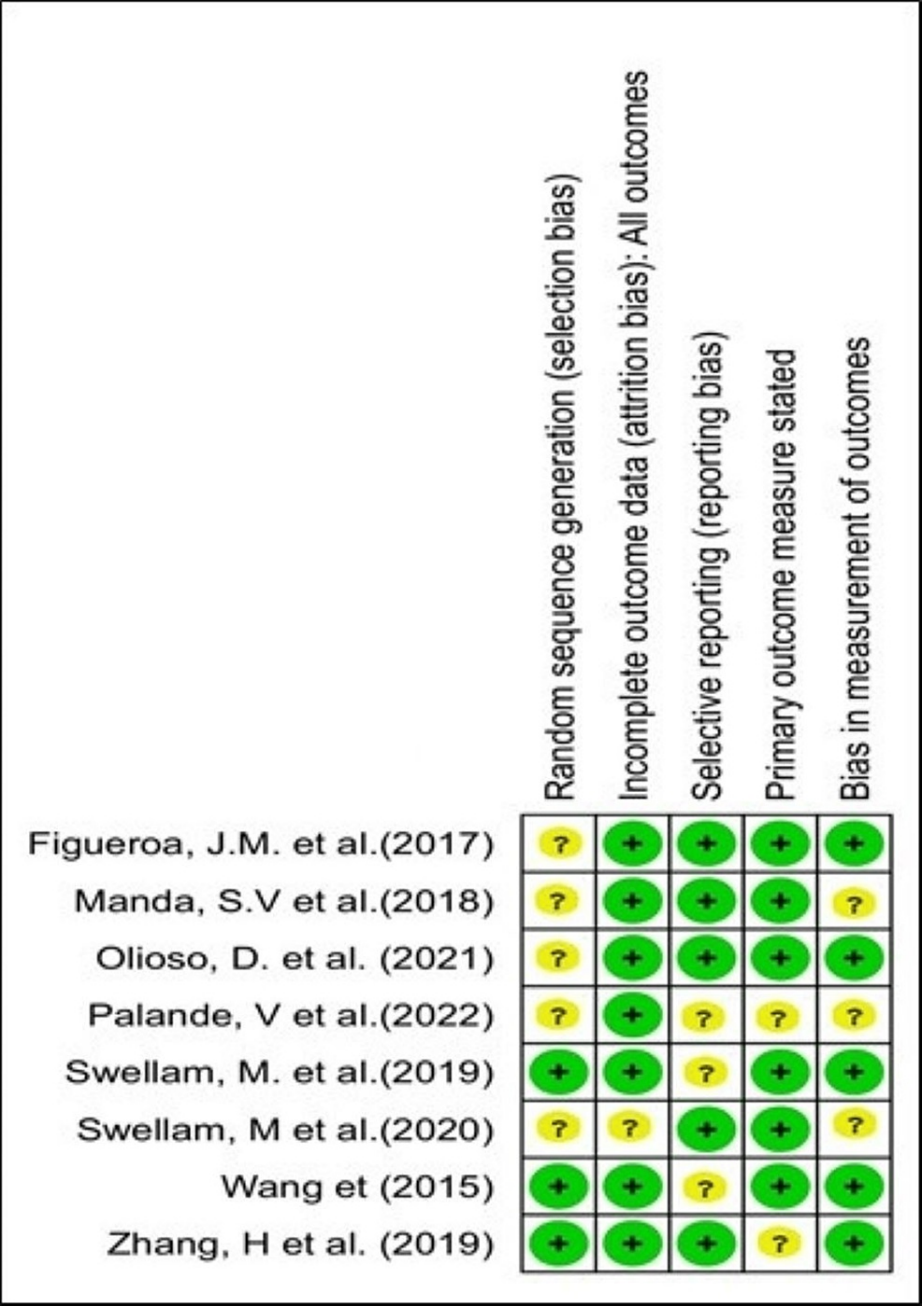
Risk of bias: graphical representation of all the incorporated studies References: [15-22]

**Figure 3:**
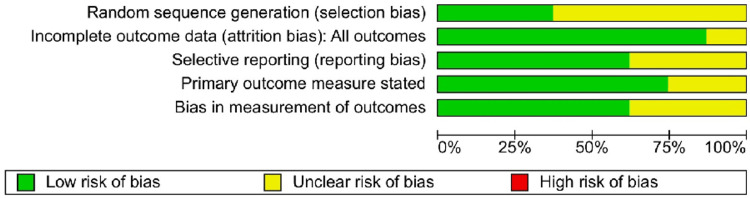
Risk of bias: overall percentage of involved studies

We employed RevMan software Version 5.4 to evaluate the risk of bias in this study. This assessment encompassed the following domains: performance bias (blinding of participants and staff), attrition bias (incomplete data), selection bias (random sequence generation), selective reporting (reporting bias), as well as additional biases. We categorized each study's risk as low (+), high (-), or unclear (?), as shown in Figure 2 [15-22]. Our comprehensive bias assessment revealed notable methodological shortcomings across all studies. These included a lack of randomization, inadequate blinding of outcome assessors (65% of trials), and unclear risk (35% of trials) (Figure 3).

Discussion

Gliomas exhibit notable genetic and epigenetic diversity, pose difficulties in surveillance, and are associated with higher rates of recurrence and mortality [14]. However, because of the high infiltrating capacity of glioma cells, and despite great advancements in both radio-/chemotherapeutic and surgical protocols, therapy of HGG still remains almost ineffective [24]. One notable finding was the downregulation of serum miR-100 expression levels in GBM patients as opposed to the control group. Moreover, a significant increase in serum miR-100 levels after treatment was observed. This suggests that serum miR-100 levels might serve as a differentiating factor among GBM cases as well as normal individuals and could potentially indicate treatment reaction [25]. miRNAs can influence the promotion of sustained signaling for proliferation as well as the ability to evade growth suppressors, enhancing the expansion capacities of cancer cells [26]. At present, GBM remains a formidable therapeutic obstacle, notwithstanding progress in surgical resection methods [27,28]. Consequently, innovative treatments are imperative for tailoring the management of HGG. In this regard, there is a pressing need for a precise, dependable, repeatable, as well as non-invasive tool to describe tumor characteristics. On the other hand, the absence of expressive biomarkers poses a significant challenge in the clinical care of glioma cases. LBs hold promise as a trustworthy as well as non-invasive approach to address this issue [29].

In this review, a total of 498 cases were identified across eight studies [15-22]. The average sensitivity was 72.28% in seven of these studies, while the average specificity was 91.52% in the same seven studies (Table 1) [15-19,22]. Although prior studies have reported that ctDNA mutations were detectable in just 10% of GBM cases, recent research has shown a remarkable increase in sensitivity, with rates reaching up to 55% [30].

In a research conducted by Li and colleagues, cfDNA was achieved by commencing two different types of samplings: first is H3.3K27M mutant as well as H3 wildtype (H3WT). These specimens included H3.3K27M tumor tissues (four samples), CSF (six samples), plasma (four samples), as well as human key pediatric glioma cells. Notably, the study reported a 100% sensitivity in addition to specificity in detecting mutations in cooperation-matched diffuse midline glioma (DMG) tissue as well as CSF samples [31]. Furthermore, additional research revealed that H3K27M was detectable in the CSF as well as plasma of 88% of DMG cases, with CSF exhibiting the highest concentration of ctDNA [32].

Additionally, Nassiri and colleagues presented findings indicating that plasma-based DNA methylation profiles efficiently displayed and identified unique characteristics in differentiating intracranial tumors. These tumors frequently share cell-of-origin lineages, posing challenges for conventional imaging techniques. To accomplish this, the researchers utilized the cell-free methylated DNA immune precipitation as well as the high-throughput sequencing (cfMeDIP-seq) technique to effectively identify ctDNA originating from brain tumors [33]. Additionally, it has been established that ctDNA can traverse the blood-brain barrier (BBB) in approximately 50% of cases possessing hypothetically targetable genomic alterations. Literature indicates the feasibility of conducting a ctDNA exploration for GBM genomic describing before disturbing surgical biopsies or in cases where surgical intervention is not feasible [34].

The capacity of LB to examine tumor-related substances present in bodily fluids has led to its growing adoption across various tumor types. This method offers crucial diagnostic and prognostic insights, along with real-time updates on tumor status. When it comes to gliomas, the application of an LB holds great promise [35].

LB has enabled the finding of cfDNA in cancer cases, presenting the opportunity for its integration into clinical practice. The primary objective is to identify genetic as well as epigenetic modifications in tumors. cfDNA holds promise as a potential molecular marker symptomatic of tumor status, offering the capability for disease monitoring and distinguishing between individuals without tumors and those with brain tumors [36].

In a precise study, control subjects consistently exhibited low levels of cfDNA in their serum, while individuals with GBM had notably advanced cfDNA concentrations. This observation suggests that cfDNA could serve as a valuable biomarker for discerning GBM cases from vigorous characters in addition to a display of tumor progression [37].

cfDNA also holds promise for quantitatively measuring genetic alterations such as isocitrate dehydrogenase (IDH) mutations and epigenetic changes like MGMT methylation [36,38]. In cfDNA resulting from glioma cases, numerous genes as well as epigenetic modifications can be identified as well as utilized as diagnostic and monitoring biomarkers for the disease. Notably, the H3K27M mutation, a hallmark of DMG, serves as a prominent example [14].

LB is capable of identifying circulating cells originating from gliomas and detecting tumor-specific genomic alterations in the bloodstream. This provides valuable insights into both local and systemic tumor responses. Using biomarker panels is recommended for improved accuracy, particularly in aggressive tumors, aiding glioma grading through multi-biosource as well as multi-biomolecule blood tests.

Limitations

Key limitations include the need to bank glioma patients' blood and standardized methods for result reproducibility. Research on the clinical utility of bloodstream-based biomarkers is ongoing, exploring alternative biofluids like CSF, urine, or saliva. Integrating various detection methods may help identify tumor characteristics and patient prognosis. However, cumulative analysis is challenging due to resource limitations.

## Conclusions

LB is a significant innovation in the field of precision medicine and has made a competent potential in the treatment of cancer; this revolutionary non-invasive technique consists of the detection and isolation of ctDNA, CT cells, and exosomes, as a source of genomic and proteomic information in cancer patients. Biomarker analysis offers a good range of information that allows non-invasive diagnosis and supports the prognosis and treatment of cancer.

Based on the findings of the current review, we observed a sensitivity of 72.28% and a specificity of 91.52%. Thus, LB can serve as a prognostic tool for the detection of CT cells. Nevertheless, the early detection of tumor cells and the prediction of tumor behavior in gliomas remain subjects of controversy. The process of biofluid collection, choice of biomarkers, and detection strategy need to be extensively studied in future clinical trials in order to allow for the incorporation of LB into clinical practice.
